# Evaluating an audit and feedback intervention for reducing antibiotic prescribing behaviour in general dental practice (the RAPiD trial): a partial factorial cluster randomised trial protocol

**DOI:** 10.1186/1748-5908-9-50

**Published:** 2014-04-24

**Authors:** Maria Prior, Paula Elouafkaoui, Andrew Elders, Linda Young, Eilidh M Duncan, Rumana Newlands, Jan E Clarkson, Craig R Ramsay

**Affiliations:** 1 Health Services Research Unit, University of Aberdeen, Health Sciences Building, Foresterhill, Aberdeen, UK; 2 Dental Health Services Research Unit, University of Dundee, Park Place, Dundee, UK; 3 NHS Education for Scotland, Dundee Dental Education Centre, Frankland Building, Dundee, UK

**Keywords:** Prescribing, Antibiotics, Dental

## Abstract

**Background:**

Antibiotic prescribing in dentistry accounts for 9% of total antibiotic prescriptions in Scottish primary care. The Scottish Dental Clinical Effectiveness Programme (SDCEP) published guidance in April 2008 (2nd edition, August 2011) for Drug Prescribing in Dentistry, which aims to assist dentists to make evidence-based antibiotic prescribing decisions. However, wide variation in prescribing persists and the overall use of antibiotics is increasing.

**Methods:**

RAPiD is a 12-month partial factorial cluster randomised trial conducted in NHS General Dental Practices across Scotland. Its aim is to compare the effectiveness of individualised audit and feedback (A&F) strategies for the translation into practice of SDCEP recommendations on antibiotic prescribing. The trial uses routinely collected electronic healthcare data in five aspects of its design in order to: identify the study population; apply eligibility criteria; carry out stratified randomisation; generate the trial intervention; analyse trial outcomes.

Eligibility was determined on contract status and a minimum level of recent NHS treatment provision. All eligible dental practices in Scotland were simultaneously randomised at baseline either to current audit practice or to an intervention group. Randomisation was stratified by single-handed/multi-handed practices. General dental practitioners (GDPs) working at intervention practices will receive individualised graphical representations of their antibiotic prescribing rate from the previous 14 months at baseline and an update at six months. GDPs could not be blinded to their practice allocation. Intervention practices were further randomised using a factorial design to receive feedback with or without: a health board comparator; a supplementary text-based intervention; additional feedback at nine months. The primary outcome is the total antibiotic prescribing rate per 100 courses of treatment over the year following delivery of the baseline intervention.

A concurrent qualitative process evaluation will apply theory-based approaches using the Consolidated Framework for Implementation Research to explore the acceptability of the interventions and the Theoretical Domains Framework to identify barriers and enablers to evidence-based antibiotic prescribing behaviour by GDPs.

**Discussion:**

RAPiD will provide a robust evaluation of A&F in dentistry in Scotland. It also demonstrates that linked administrative datasets have the potential to be used efficiently and effectively across all stages of an randomised controlled trial.

**Trial registration:**

Current Controlled Trials ISRCTN49204710

## Background

The Scottish Dental Clinical Effectiveness Programme (SDCEP) was established in 2004 as a joint initiative of the National Dental Advisory Committee and NHS Education for Scotland (NES) with the aim of providing user-friendly evidence-based guidance in priority areas for dental healthcare in Scotland. The guidance it produces is designed to assist dental teams and to improve patient health by presenting advice and recommendations that are based on the best available information, thereby facilitating the translation of research evidence into practice and assisting in compliance to the ever changing regulatory framework of healthcare provision.

In April 2008, SDCEP published guidance for Drug Prescribing in Dentistry that is suitable for informing dental practitioners in the primary care sector, and applies to all patients who would be treated in the primary care sector in Scotland [1]. The guidance brought together advice and recommendations on dental prescribing from the British National Formulary (BNF) and the BNF for Children and from the National Institute for Health and Clinical Excellence and presented it in a readily accessible, problem orientated style. In August 2011, a second edition of Drug Prescribing for Dentistry was published by SDCEP. In addition, a ‘dental prescribing’ app for iPhone®, iPad®, and iPod touch® was launched in April 2012 [2].

Despite the introduction of the SDCEP guidance, there is wide variation in dental drug prescribing in Scotland. Antibiotic prescribing in dentistry accounts for 9% of total antibiotic prescribing in primary care in Scotland. In 2011, the overall use of antibiotics in dentistry was reported to be 7.6% higher than in 2010 [3]. General dental practitioners (GDPs) prescribe antibiotics regularly for the management of dental infections, but treatment is often guided by personal experience and knowledge [4,5], and in many cases antibiotics are prescribed inappropriately to patients with dental emergencies [6]. It is widely recognised that antimicrobial resistance is a major threat to public health and patient safety. Inappropriate antibiotic prescribing contributes to an increasing risk of antimicrobial resistance [3].

In dentistry, there is ample evidence that the implementation of guidance by dental professionals is variable and understanding how to change this is limited [7]. However, there is a lack of robust, generalisable evidence on how best to promote the translation of guidance into practice. This was highlighted in the Health Technology Assessment (HTA) systematic review of the effectiveness and efficiency of guideline dissemination and implementation strategies [8], which found evidence that although dissemination and implementation of guidance could lead to compliance with recommendations, the effect varied both within and across interventions.

Audit and Feedback (A&F) is defined as ‘any summary of clinical performance of healthcare over a specified period of time’ aimed at changing health professional behaviour [9]. A&F is a commonly used approach and has consistently demonstrated small to moderate sized effects, [9] though predicting the clinical circumstances where effects will be moderate and those where effects are small has been elusive [10].

In Scotland, routinely collected electronic healthcare data (*e.g.*, number of claims for treatment and number of prescription items dispensed) are available at the level of the individual GDP. One way in which current practice may be enhanced is by the provision of individualised feedback on antibiotic prescribing to GDPs. Another way is with the addition of behaviour change techniques (BCTs). A BCT is a ‘systematic procedure included as an active component of an intervention designed to change behaviour’ [11]. BCTs are the ‘active ingredients’ in an intervention (*i.e.*, they are the proposed mechanisms of change). Guidelines tend to be lengthy, and it is unknown whether providing clinicians with a distillation of the guidelines solely into the BCTs could be an advantageous way of enhancing implementation and changing behaviour.

The RAPiD (Reducing Antibiotic Prescribing in Dentistry) trial will compare the effectiveness of individualised A&F strategies for the translation into practice of SDCEP recommendations on antibiotic prescribing. The objectives of the trial relate to antibiotic prescribing behaviour at dental practice level. The research question being addressed is: In comparison to current practice, does the provision of individualised feedback on antibiotic prescribing at differing intervals (*i.e.*, 0 and 6 months ± 9 months), with or without a comparator, with or without a text-based intervention reiterating the ‘active ingredients’ within the SDCEP recommendations on antibiotic prescribing lead to a reduction in antibiotic prescribing in dental primary care in Scotland?

In addition, a process evaluation will explore the acceptability of the individualised A&F strategies and identify barriers and enablers to evidence-based antibiotic prescribing practice in dentistry. The RAPiD trial is being conducted as part of NES’s TRiaDS (Translation Research in a Dental Setting) programme [12]. At writing (December 2013), data collection and delivery of the individualised baseline and six months A&F interventions have taken place.

## Methods

### Trial Design

**Figure 1:**
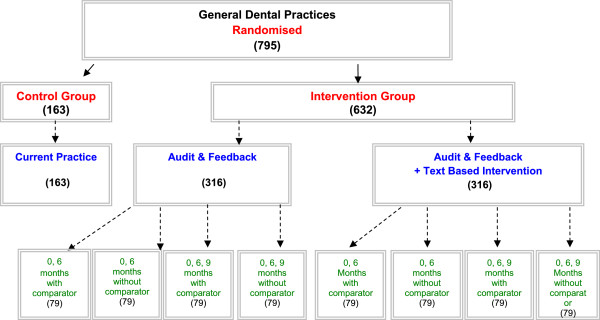
Study design.

The RAPiD trial is a 12-month partial factorial cluster randomised controlled trial conducted in NHS General Dental Practices across Scotland. A cluster design was used to reduce contamination within dental practices. A factorial design was used to assess the effect of the three A&F strategies (*i.e.*, inclusion of a comparator, inclusion of a text-based intervention and the interval between receiving feedback). We will also explore the effects of the interventions combined. A control group was included to test the effectiveness of any form of A&F strategy (Figure 1).

The trial uses routinely collected electronic healthcare data in five aspects of the trial design in order to: identify the study population; apply eligibility criteria; carry out stratified randomisation; generate individualised feedback for the trial intervention; and analyse trial outcomes.

Permission was granted by the NHS National Services Scotland (NSS) Privacy Advisory Committee (PAC Ref: 10/12) to link data from the Management and Dental Accounting System (MIDAS) database with the Prescribing Information System for Scotland (PRISMS) database.

### Initial data linkage and processing

Information Services Division (ISD) of NHS National Services Scotland (NSS) holds the MIDAS and PRISMS databases. The MIDAS database contains claims information relating to all courses of NHS dental treatment provided by GDPs in the General Dental Service since 1990. The PRISMS database contains information for all primary care prescriptions dispensed in community pharmacies (including dental prescriptions) since April 2004.

ISD supplied the TRiaDS Programme Office with the treatment data extracted from MIDAS and with monthly dental prescribing PRISMS data. The data from MIDAS and PRISMS were linked to a further extract from MIDAS containing contact information for GDPs and dental practices across Scotland. This extract was supplied by the Practitioner Services Division (PSD) of NHS NSS and listed all GDPs practising as at April 2013.

The GDP list number was used as the single common identifier. The list number is common to both MIDAS and PRISMS (in PRISMS the list number is used as the Prescriber ID) and is a unique number issued to GDPs by Health Boards in order for them to submit item-of-service and registration claims for payment. The linked dataset does not contain any patient identifiable information and was processed in accordance with the Data Protection Act 1998.

### Intervention development

#### Individualised audit and feedback interventions

Monthly prescribing volume was determined by the number of antibiotic items (BNF section 5.1) prescribed in the General Dental Service and subsequently dispensed each month. The monthly number of claims was determined as the number of ordinary list claims for treatment recorded in MIDAS each month (*i.e.*, claims made for NHS treatment carried out for patients registered under a dentist’s standard list number at a given location). Claims made on other lists, *e.g.*, emergency, trainer, and assistant were excluded. After the six-month intervention was delivered, the status of some ordinary lists in MIDAS was reclassified as public dental service and these will be included thereafter. For each individual GDP, the prescribing rate was calculated as the monthly number of items dispensed divided by the mean monthly number of claims (multiplied by 100). The initial feedback contained retrospective prescribing rates taken from the previous 14 months (November 2011 to December 2012). Monthly prescribing rates for health boards were similarly calculated based on total antibiotic items prescribed and total number of treatment claims within each health board.

### Development of the text based intervention

The sections on bacterial infections, in the published SDCEP clinical guidance on ‘Drug Prescribing for Dentistry’, were coded for the presence/absence of BCTs using the 2012 BCT taxonomy [13,14]. Two BCTs were identified: instruction on how to perform the behaviour; and provide information about health consequences of performing the behaviour. These BCTs were therefore selected for inclusion within a text-based intervention. The SDCEP guidance included behavioural instruction relating to pre-decision processes, *i.e.*, whether or not it is appropriate to prescribe antibiotics; and post-decision processes, *i.e.*, ways to optimise antibiotic prescribing once the decision to prescribe had been made. We chose to include only the BCTs that focussed on the pre-decision processes. Where possible, the exact wording from the SDCEP guidance document was used. A number of potential BCTs coded in the guidance had to be excluded from the final text-based intervention due to insufficient specification of behaviour. For example, the behaviour ‘take care’ within ‘take care when prescribing these antibiotics to vulnerable groups’ was not explicit enough to be included as ‘instruction on how to perform the behaviour’. The wording of text-based intervention used in the trial is:

‘Prescribing courses of antibiotic treatment can encourage the development of antimicrobial resistance and therefore must be kept to a minimum’.

‘As a first step in the treatment of bacterial infections, use local measures. For example, drain pus if present in dental abscesses by extraction of the tooth or through root canals, and attempt to drain any soft-tissue pus by incision’.

‘This should be the first step even if patients request antibiotics and even when time is short’.

‘Antibiotics are appropriate for oral infections where there is evidence of spreading infection, systemic involvement or persistent swelling despite local treatment’.

‘Use antibiotics in conjunction with, and not as an alternative to, local measures’.

The development of the text-based intervention is reported in more detail in Additional file 1.

### Study population and eligibility

Practice addresses and the names of all GDPs working in NHS General Dental Practices across the 14 Scottish Health Boards were identified from the April 2013 PSD GDP list.

### Dental practice inclusion criterion

NHS General Dental Practices across the 14 Health Boards in Scotland.

### Dental practice exclusion criteria

Practices in mainland Health Boards in which any GDPs were salaried (Salaried GDPs are used as a proxy to identify community and emergency dental services). Predominantly due to geography, the majority of dental services in the Island Health Boards are provided by the NHS salaried service. For this reason, practices with salaried GDPs in the Island Boards are not excluded).

General Dental Practices where no ordinary list claims were made in more than 6 months out of the most recently available 12 months of MIDAS data at the time of the baseline intervention (January to December 2012).

### GDPs inclusion criteria

For the baseline intervention, GDPs listed by PSD as currently practicing in April 2013.

For the six-month intervention, GDPs listed by PSD as currently practicing in October 2013.

For the nine-month intervention, GDPs listed by PSD as currently practicing in January 2014.

### GDPs exclusion criterion

GDPs with no name recorded in the PSD list.

### Randomisation and allocation

All eligible practices (n = 795) were simultaneously randomised by AE at the beginning of the trial before any baseline feedback data were issued. The allocation schedule for random assignment was computer generated. Practices were ordered randomly, with the first 632 practices being allocated to an A&F intervention and the remaining practices being allocated to the control group. Each of the 632 intervention practices was allocated to one of the eight subgroups (see Figure 1) with an even allocation so that 79 practices were randomised to each subgroup. Randomisation was also be stratified by single-handed/multi-handed practices.

### Initial and follow-up procedures

**Figure 2:**
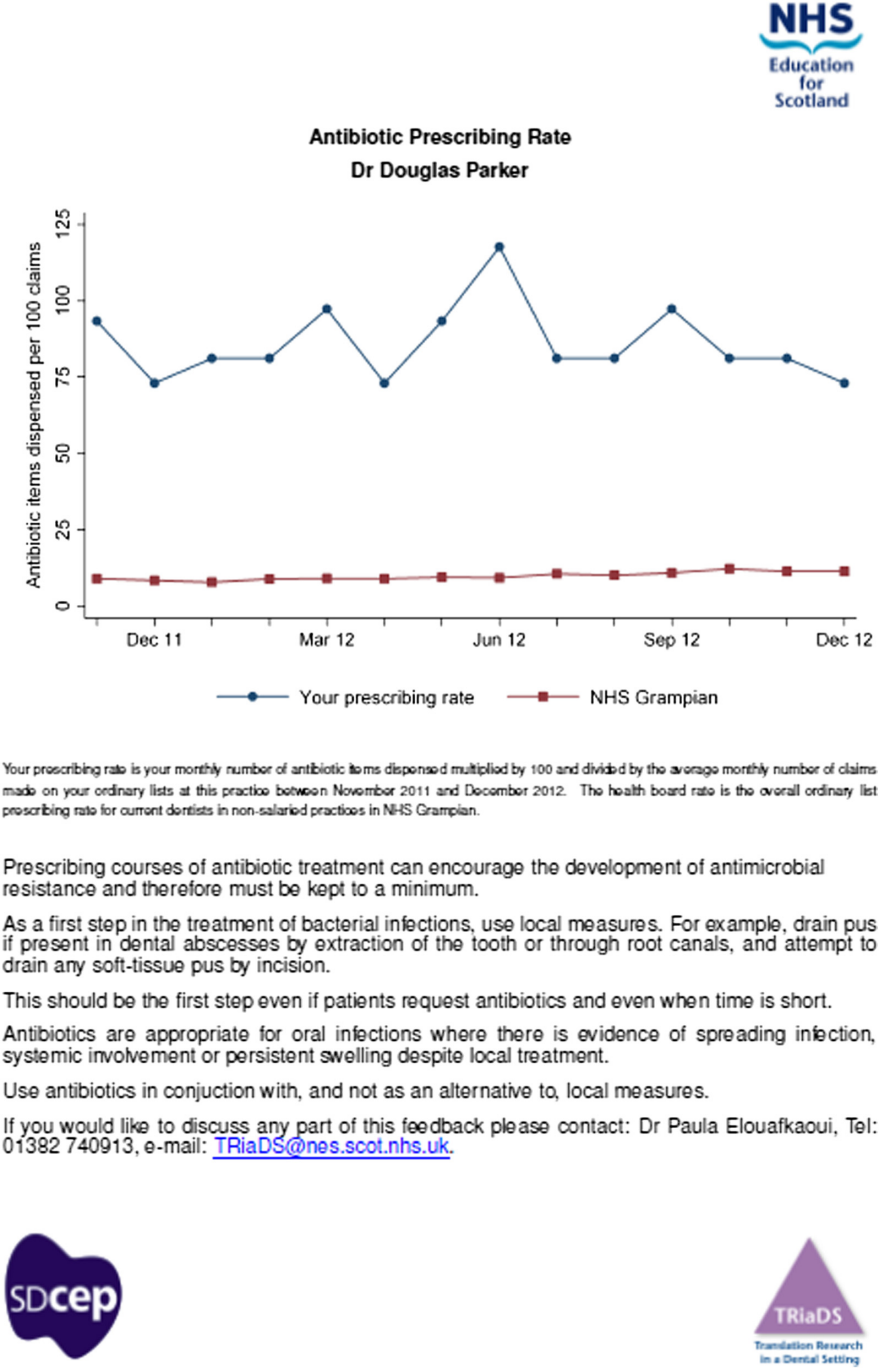
Example feedback intervention document (anonymised with pseudonym).

All GDPs working in the 632 practices allocated to an intervention group received feedback (*i.e.*, feedback on antibiotic prescribing ± a comparator, ± a text based intervention) at baseline (May 2013) and updated feedback at six months (Nov 2013) (Figure 1). At nine months (February 2014), a further round of feedback will be delivered to GDPs in 50% of practices allocated to an intervention group (Figure 1). All intervention documents will be in paper form and delivered by post (Figure 2). These will be accompanied by a personalised cover letter providing information about the study and contact details for the TRiaDS Programme Office in the event of questions or requests for further information (Additional file 2).

The feedback at six months is in the same format as the baseline feedback but contains a further six months of prescribing rate data in addition to the 14 months of data fed back at baseline. The nine-month feedback will again be of a similar format. Additional PRISMS and MIDAS data were obtained from ISD to include data up to June 2013 for the six-month feedback and will be obtained to September 2013 for the nine-month feedback. The linked data relating to the 795 included practices from these updated datasets will also be linked to the list of eligible GDPs published by PSD (in October 2013 for the six-month update, in January 2014 for the nine-month update). Updated feedback sheets will be sent to GDPs at practices randomised to the intervention group. It is recognised that some GDPs and practices may be lost to follow-up (*e.g.*, if a practice closes or a GDP stops practising in Scotland).

### Outcomes

All outcomes pertain to cluster level (*i.e.*, dental practices).

The primary outcome is the total number of antibiotic items (from Section 5.1 of the British National Formulary [15]) dispensed per 100 claims over the 12 months from May 2013 to April 2014.

The secondary outcomes are:

1. Total number of Amoxicillin 3 g items dispensed per 100 claims over the 12 months from May 2013 to April 2014 [16].

2. Total number of ‘second-line’ antibiotic items (Clindamycin, Co-amoxiclav, Clarithromycin) [1] dispensed per 100 claims over the 12 months from May 2013 to April 2014.

3. Total defined daily doses of antibiotics [17] (from Section 5.1 of the British National Formulary [15]) dispensed per 100 claims over the 12 months from May 2013 to April 2014.

4. Total defined daily doses of Amoxicillin 3 g dispensed per 100 claims over the 12 months from May 2013 to April 2014.

5. Total defined daily doses of ‘second-line’ antibiotics (Clindamycin, Co-amoxiclav, Clarithromycin) [1] dispensed per 100 claims over the 12 months from May 2013 to April 2014.

### Data collection and processing

#### Schedule of data collection

MIDAS and PRISMS data are updated routinely by ISD on a monthly basis with a time lag of between two and three months. Updates of data will be received by the TRiaDS Office every month throughout the duration of the trial. Data covering the period up to 31 December 2012 was received in March 2013 and used for the intervention at baseline. Data up to 30 June 2013 was received in September 2013 and used for the six-month intervention (feedback was disseminated on 1 November 2013). Similarly, data up to 30 September 2013 will be used for the nine-month intervention (feedback will be disseminated on 1 February 2014).

### Analysis plans

#### Statistical analysis

Outcomes will be aggregated and analysed at the practice level. The analysis will estimate the effect of each intervention compared with current practice and will also estimate the differential effect between interventions (*i.e.*, inclusion of a text-based intervention, frequency of feedback and inclusion of a comparator). We will perform main effects analyses of covariance on the 12-month prescribing rate, adjusting for the pre-intervention yearly prescribing rate, practice size (single-handed/multi-handed), and the annual number of treatment claims submitted by the practice. The model fitting strategy will test the practice size variable (design), then the pre-intervention prescribing rate (covariate), and lastly a categorical variable (intervention) will be included to correspond with the main effects of the different interventions. The same approach will be used for all prescribing behaviours under investigation.

We will investigate whether larger effects of the interventions are observed for practices reporting higher pre-intervention levels of prescribing (defined as upper quartile practices). Additionally, to test for a reduction in the spread of prescribing levels, we will apply Levene’s test for equality of variances between the pre-intervention and intervention phases.

### Timing and frequency of analysis

A single principle analysis is anticipated at 12 months post baseline intervention (*i.e.*, covering up to 30 April 2014). The data will be available in July 2014 and will be used to measure all outcomes.

### Sample size

The sample size calculation is based on aggregated practice level antibiotic prescribing. There will be 795 practices participating in the trial, of which 632 practices will be randomised to the intervention group. Each of the eight sub-level experimental units in the intervention group will have 79 practices. This is the required sample size to achieve 80% power (with two-sided alpha of 2.5% allowing for multiple comparisons) to detect a 10% difference in overall antibiotic prescribing between intervention groups. This applies to the comparison between A&F only (n = 316) and A&F with a text-based intervention (n = 316), the comparison between feedback at zero, six, and nine months versus zero and six months only and the comparison between those with and without a Health Board comparator. The sample size has been reduced using the correction described by Borm *et al*. for trials with correlated data [18]. The comparison between the control group (n = 163) and the intervention group (n = 632) will have 80% power to detect a 12% decrease in overall antibiotic prescribing.

The sample size calculation is based on prescribing activity recorded in PRISMS for the 1,799 dentist lists in Scotland known to be prescribing throughout the year ending June 2010. The mean number of antibiotic items prescribed per list was 141.1 with an SD of 140.9. The correlation with the year ending June 2009 is 0.91.

### Process evaluation

The 2008 Medical Research Council guidance for developing complex interventions [19] proposes the use of evidence and theory to develop an understanding of the likely processes of change. In order to facilitate understanding of the processes associated with antibiotic prescribing in dentistry, we will conduct a concurrent theoretically informed process evaluation using the Theoretical Domains Framework (TDF) [20,21] from health psychology and the Consolidated Framework for Implementation Research (CFIR) [22]. The TDF is built from 33 behavioural theories, and proposes that determinants of healthcare professionals’ behaviour cluster into 14 ‘domains’ (*e.g.*, beliefs about consequences, social influences, professional role and identity) [21]. It allows for consideration of a comprehensive range of potential influences on health professional behaviour and evidence suggests that TDF-based interviews may prompt respondents to identify barriers that they would not otherwise report [23]. In this study, the behaviour of interest is managing dental patients with bacterial infections using local measures rather than prescribing antibiotics. The CFIR consists of common constructs from published implementation theories and offers an over-arching typology to promote implementation theory development and verification to understand the mechanism about what works, where, and why across various contexts [22].

The process evaluation will involve semi-structured telephone interviews, with up to 30 GDPs working within eligible dental practices (*i.e.*, practices allocated to an A&F intervention group or allocated to the control group). The aims of the process evaluation are to: identify barriers and enablers to evidence-based antibiotic prescribing behaviour by GDPs (*i.e.*, barriers and enablers to managing patients with bacterial infections using local measures rather than prescribing antibiotics); and explore GDPs’ experiences of and response to the individualised A&F interventions.

### Sampling and recruitment

Potential participants will be identified from the linked MIDAS and PRISMS data. All GDPs included in the RAPiD trial (control and intervention groups) will be categorised into three groups (low, medium, and high) according to their antibiotic prescribing level during the period covered by the baseline intervention. Low prescribers are those whose prescribing rate was in the lower quartile in every month and high prescribers are those whose prescribing rate was in the upper quartile in every month. The remaining GDPs will be categorised as medium prescribers. Three hundred potential participants (100 low prescribers, 100 medium, and 100 high) will be sampled using implicit stratification ensuring representativeness based on the following baseline factors: Health Board; practice prescribing profile (*e.g.*, all GDPs in the practice are high/medium/low prescribers or a mixture of high/medium/low prescribers); practice size (*i.e.*, single/multi-handed).

A purposive sample of between 15 and 30 GDPs will be interviewed (*e.g.*, up to 12 GDPs from each of the two main intervention groups plus six from the control group). Recruitment will commence after the six-month feedback in the RAPiD trial (January 2014) and will continue until 30 GDPs have been interviewed, or until data saturation occurs across the theoretical domains (*i.e.*, no new beliefs are being introduced within the 14 TDF domains) [24]. Throughout recruitment, diversity variables will be tracked in order to inform ongoing sampling of potential participants and maximise representativeness in terms of: individual prescribing level (low/medium/high antibiotic prescribers); practice prescribing profile; Health Board; practice size; and rurality and deprivation. Rurality will be measured by the urban/rural classification [25] and deprivation by the Scottish Index of Multiple Deprivation (SIMD) category [26], with both measures based on the practice postcode.

A letter of invitation to take part in the interview study (with an option to opt out of being contacted further) will be sent initially to 100 of the 300 potential participants representative on the diversity variables of interest (*e.g.*, prescribing level, health board) and on intervention/control allocation. GDPs who contacted the TRiaDS Office after receiving their intervention materials will also be included in this initial batch of invitations. As an incentive, GDPs who take part in the interview study will be eligible to claim a payment of £40. The response rate from the initial mailing will determine the need to send letters of invitation to the remaining 200 GDPs in the stratified sample. That is to say, if recruitment from the initial 100 letters fails to result in 30 interviews, a further 100 letters will be sent. The process will be repeated a third time if necessary in order to achieve a sample of 30 GDPs.

Within two weeks of the initial mailing, GDPs who do not opt out will be contacted by a member of the research team, by telephone, to discuss the study and to ascertain their willingness to take part. A mutually convenient time for a telephone interview will be arranged with those GDPs expressing an interest in taking part. No further contact will be made with dentists who do not wish to be interviewed. Informed verbal (recorded) consent will be obtained from all participants prior to interview.

### Data collection and analysis

A semi-structured topic guide has been developed based on the TDF and CFIR to address the aims of the process evaluation (Additional file 3). Telephone interviews will last approximately 30 minutes and, with the participants’ permission, will be audio-recorded and transcribed verbatim. Telephone interviews are considered more appropriate than face-to-face interviews for this study. This decision relates both to attempts to minimise potential burden for participants and to the efficient use of resources (*i.e.*, time, cost, effort) for generating data on this national sample.

The transcribed interviews will be content analysed in NVivo using the TDF and CFIR as coding frameworks [22,27]. Data will be analysed throughout recruitment on an on-going basis until a sample size of 30 is achieved, or the point of saturation (where no new responses emerge) is reached [24]. In addition to identifying specific beliefs relating to prescribing behaviour, the analyses will aim to determine which domains could be potential intervention targets [27]. There will be research team discussion throughout analysis process to raise multiple perspectives, and decision will be made through a process of deliberation and consensus.

### Ethical considerations and authorisations

All members of the RAPiD trial study team have undergone training in Good Clinical Practice (GCP). GCP is an international ethical and scientific quality standard for designing, conducting, recording, and reporting studies that involve human participants.

In September 2011, a harmonised edition of the UK Governance Arrangements for Research Ethics Committees (GAfREC) came into effect and research involving NHS staff as participants by virtue of their professional role was excluded from the normal remit of NHS Research Ethics Committees. The East of Scotland Research Ethics Service considered the RAPiD trial protocol and confirmed that it did not require ethical review or approval by an NHS REC (Ref: 11/GA/229). In addition, the protocol was submitted to the NHS Research Scotland Permissions Coordinating Centre and reviewed by the Tayside Medical Science Centre (TASC) Research and Development (R&D) office. They classified the RAPiD trial as service development/audit and confirmed that it did not require R&D registration, formal review, or approval. Confirmation has been received from all 14 Scottish Health Boards that they have been notified about the trial and have added it to their Audit and Clinical Governance records. The RAPiD trial’s International Standard Randomised Controlled Trial Number (ISRCTN) registration is ISRCTN 49204710.

### Trial status

To date (December 2013), baseline and six-month feedback interventions have been delivered.

## Discussion

The RAPiD trial has been designed to test the effectiveness of individualised A&F strategies for the implementation of evidence-based guidance on antibiotic prescribing in real world dental practice. The trial is being conducted as part of the TRiaDS programme of multi-disciplinary implementation research embedded within the SDCEP guidance development process [12]. TRiaDS and SDCEP have a national, education, and service remit to support the development and implementation of national clinical guidance addressing quality improvement and patient safety priorities for dental healthcare in Scotland. The Programmes’ collaborative links with dental healthcare policy makers and providers have ensured the research question addressed meets the needs of the service and have increased the likelihood of the most effective intervention being adopted as part of routine service delivery [12].

The study is innovative in its use of routinely collected electronic healthcare data across all stages of the trial design. In particular, administrative datasets held by ISD (MIDAS and PRISMS) are linked to: identify the study population; apply eligibility criteria; carry out stratified randomisation; generate individualised feedback for the trial intervention; and analyse trial outcomes. Methodological strengths of this design include: minimisation of assessment reactivity (*e.g.*, non-contact recruitment of trial participants, no contact (postal) delivery of the trial intervention; and no self-report measures); no opportunity for researchers to influence the antibiotic prescribing rates presented in the feedback [28]. These features reduce the potential for pre- and post-randomisation sources of bias associated with recruitment, baseline assessment activities, exposure to study conditions, and to assessment at follow-up [28]. Another methodological strength of the RAPiD trial is that it operationalises published recommendations for the design of A&F intervention studies [10]. Specifically, RAPiD adopts ‘best practices’ for A&F components (*e.g.*, data are individualised, based on recent performance and new data are presented over time), investigates further optimisation strategies in conjunction with a process evaluation and applies relevant theory to the development of the text-based intervention [10].

The use of administrative datasets presents limitations as well as strengths. For example, PRISMS collects dispensing rather than prescribing data and MIDAS is a repository for remuneration data rather than treatment provided. Claims for payment for dental treatment are submitted to MIDAS at the end of a course of treatment. In some instances, a course of treatment may be delivered over a number of weeks, while an antibiotic may be prescribed and dispensed at any time during this period. Thus, only a proxy measure of the rate of antibiotic prescribing can be obtained from these datasets.

The SDCEP guidance for Drug Prescribing in Dentistry states that it is appropriate to prescribe antibiotics ‘if local measures prove ineffective or if there is evidence of cellulitis, spreading infection or systemic involvement’ [1]. However, a limitation of this study is that it is not possible to determine, from routine data, whether or not antibiotics have been prescribed appropriately. Another limitation relates to the three-month time lag between data being collected by ISD and being made available on PRISMS and MIDAS.

Evidence suggests that greater effectiveness is shown with increased frequency of feedback [29]. In the RAPiD trial we tested two feedback frequencies. However, in the intervention group with feedback delivered more frequently (zero, six, and nine months) it was not appropriate to deliver the A&F strategies at three months after baseline, because the graphical feedback would not have included any post-baseline data.

In summary, the partial factorial cluster trial design and the theory-based qualitative process evaluation will provide a robust evaluation of A&F in dentistry in Scotland and will help elucidate the mechanisms by which this approach works with a view to maximising applicability to other settings.

## Abbreviations

A & F: Audit and Feedback; BCTs: Behavioural change techniques; BNF: British national formulary; CFIR: Consolidated framework for implementation research; GDPs: General dental practitioners; ISD: Information services division; MIDAS: Management and dental accounting system; NHS: National health services; NSS: NHS National services Scotland; PRISMS: Prescribing information system for Scotland; PSD: Practitioner services division; RAPiD: Reducing antibiotic prescribing in dentistry; SAPG: Scottish antimicrobial prescribing group; SDCEP: Scottish dental clinical effectiveness programme; TDF: Theoretical domains framework; TRiaDS: Translation research in a dental setting.

## Competing interests

The authors declare they have no competing interests.

## Authors’ contributions

All authors contributed to the conceptual and theoretical development of the study. PE and AE conducted the data linkage. MP drafted the manuscript. All authors critically reviewed and contributed to draft revisions, and read and approved the final version of the manuscript.

## Supplementary Material

Additional file 1 Development of the text-based intervention. Click here for file

Additional file 2 RAPiD Process Evaluation Interview Topic Guide. Click here for file

Additional file 3: Table S1 CONSORT 2010 checklist of information to include when reporting a cluster randomised trial. Click here for file
