# Supplementary material for: Evaluating an audit and feedback intervention for reducing antibiotic prescribing behaviour in general dental practice (the RAPiD trial): a partial factorial cluster randomised trial protocol
Source: Implement Sci. 2014 Apr 24;9:50. doi: 10.1186/1748-5908-9-50 (PMC4108126; doi:10.1186/1748-5908-9-50)
Supplement: Additional file 1 — Development of the text-based intervention. [file 1748-5908-9-50-S1.pdf]

Additional File 1 - Development of the text-based intervention

Final text based intervention:

Prescribing courses of antibiotic treatment can encourage the development of antimicrobial resistance and therefore must be kept to a minimum.

As a first step in the treatment of bacterial infections, use local measures. For example, drain pus if present in dental abscesses by extraction of the tooth or through root canals, and attempt to drain any soft-tissue pus by incision.

This should be the first step even if patients request antibiotics and even when time is short.

Antibiotics are appropriate for oral infections where there is evidence of spreading infection, systemic involvement or persistent swelling despite local treatment.

Use antibiotics in conjunction with, and not as an alternative to, local measures.

Development of text-based intervention

The published SDCEP guidance on antibiotic prescribing was coded for the presence/absence of BCTs using the 2012 BCT taxonomy [13,14] by ED.

**Content**<br><br>**Based on Scottish Dental Clinical Effectiveness Programme – *Drug Prescribing for Dentistry: Dental Clinical Guidance*, 2nd Edition**	**Underlying Behaviour Change Technique**<br><br>**Based on** Michie, S., Richardson, M., Johnston, M., Abraham, C., Francis, J., Hardeman, W., Eccles, M. (under review). The Behaviour Change Technique Taxonomy (v1) of 93 hierarchically-clustered techniques: building an international consensus for the reporting of behavior change Interventions.	**Inclusion/exclusion**
Prescribing prolonged courses of antibiotic treatment can encourage the development of drug resistance	78. Provide information about health consequences of performing the behaviour (note: consequences can be for any target, not just the recipient of the intervention)	Included with minor changes to ensure directly relevant for dentists i.e. ‘prolonged’ removed as dentists are unlikely to be prescribing prolonged courses, ‘drug resistance’ changed to ‘antimicrobial

		resistance'.
The use of broad-spectrum antibiotics has been associated with the rise in *Clostridium difficile*-associated disease	78. Provide information about health consequences of performing the behaviour (note: consequences can be for any target, not just the recipient of the intervention)	Excluded as known to all and therefore should not prioritise including this
Prescribing of antibiotics must be kept to a minimum	36. Instruction on how to perform the behaviour (advise or agree on how to perform the behaviour)	Included
Take care when prescribing these antibiotics to vulnerable groups (e.g. elderly, those with a history of gastrointestinal disease including those using proton pump inhibitor drugs for dyspepsia and gastro-oesophageal reflux disease)	36. Instruction on how to perform the behaviour (advise or agree on how to perform the behaviour)	Excluded because 'take care' too vague for behavioural instruction
As a first step in the treatment of bacterial infections, use local measures. E.g. drain pus if present in dental abscesses by extraction of the tooth or through root canals, and attempt to drain any soft-tissue pus by incision.	36. Instruction on how to perform the behaviour (advise or agree on how to perform the behaviour)	Included
It is appropriate to prescribe antibiotics for oral infections where there is evidence of spreading infection (cellulitis, lymph node involvement, swelling) or systemic involvement (fever, malaise)	36. Instruction on how to perform the behaviour (advise or agree on how to perform the behaviour)	Included but shortened and merged with below into one sentence due to space restrictions
Other indications to prescribe antibiotics are acute necrotising ulcerative gingivitis and sinusitis, and pericoronitis where there is systemic involvement or persistent swelling despite local treatment	36. Instruction on how to perform the behaviour (advise or agree on how to perform the behaviour)	Included (shortened and merged with above due to space restrictions)
Use antibiotics in conjunction with, and not as an alternative to, local measures.	36. Instruction on how to perform the behaviour (advise or agree on how to perform the behaviour)	Included
Before prescribing antibiotics, refer to the BNF (www.bnf.org) and BNFC (www.bnfc.org) for drug interactions.	36. Instruction on how to perform the behaviour (advise or agree on how to perform the behaviour)	Excluded as relates to behaviour post-decision to prescribe antibiotics

When prescribing antibiotics, advise patients to space out doses as much as possible throughout the day.	36. Instruction on how to perform the behaviour (advise or agree on how to perform the behaviour)	Excluded as relates to behaviour post-decision to prescribe antibiotics
Review patients who have received a course of antibiotic treatment.	36. Instruction on how to perform the behaviour (advise or agree on how to perform the behaviour)	Excluded as relates to behaviour post-decision to prescribe antibiotics
This should be the first step even if patients request antibiotics and even when time is short.	N/A	Included as relates to anecdotal evidence from dentist colleagues about potential influences on antibiotic prescribing
