# Supplementary material for: Evaluating an audit and feedback intervention for reducing antibiotic prescribing behaviour in general dental practice (the RAPiD trial): a partial factorial cluster randomised trial protocol
Source: Implement Sci. 2014 Apr 24;9:50. doi: 10.1186/1748-5908-9-50 (PMC4108126; doi:10.1186/1748-5908-9-50)
Supplement: Additional file 2 — RAPiD Process Evaluation Interview Topic Guide. [file 1748-5908-9-50-S2.pdf]

Ref: «Ref»

Dundee Dental Education Centre
Frankland Building
Small's Wynd
Dundee
DD1 4HN

NHS Education for Scotland

M/260

«title» «forename» «surname»
«address1»
«address2»
«address3»
«address_4»
«address5»
«postcode»

1st May 2013

Dear «title» «surname»

Audit and Feedback for Antibiotic Prescribing in Dentistry

Dental prescribing now accounts for approximately 9% of the total number of antibiotic prescriptions in primary care in Scotland. First published in April 2008 (updated in August 2011), the Scottish Dental Clinical Effectiveness Programme's (SDCEP) Drug Prescribing for Dentistry guidance includes information to assist dentists make decisions about antibiotic prescribing. To further support the implementation of this guidance, SDCEP and the Translation Research in a Dental Setting (TRiaDS) programme are providing dentists in Scotland with individualised feedback of their antibiotic prescribing practice.

Your enclosed feedback is in graphical form and displays: 1) your monthly antibiotic prescribing rate for your ordinary list(s) within this dental practice; 2) the average (median) antibiotic prescribing rate for ordinary lists within NHS «Health_Board». Please note you have not received information about the prescribing patterns of other dentists in your practice and it is not possible for you to identify any other dentist from the feedback given. Similarly no patient identifiable information is provided.

The feedback was produced using summary data from the MIDAS database and data from the Prescribing Information System for Scotland (PRISMS). The PRISMS database contains information for all primary care prescriptions dispensed in community pharmacies over the last five years. Both databases are held centrally by Information Services Division Scotland. Permission to use summary data has been granted by the NHS National Services Scotland Privacy Advisory Committee (PAC Ref: 10/12).

If you require any further information, please do not hesitate to contact Dr Paula Elouafkaoui, Research Fellow, TRiaDS. Tel: 01382 740913, Email: TRiaDS@nes.scot.nhs.uk.

Yours sincerely,

Prof Jan Clarkson	Dr Linda Young	Dr Paula Elouafkaoui
Programme Director	Research Manager	Research Fellow
SDCEP	SDCEP	TRiaDS

SDcep

TRiaDS
Translation Research in a Dental Setting
